# The Pro12Ala polymorphism in the PPAR­γ2 gene is not associated with an increased risk of NAFLD in Iranian patients with type 2 diabetes mellitus

**DOI:** 10.1186/s11658-019-0138-0

**Published:** 2019-03-15

**Authors:** Leila Saremi, Shirin Lotfipanah, Masumeh Mohammadi, Hassan Hosseinzadeh, Mina Fathi-Kazerooni, Behrooz Johari, Zohreh Saltanatpour

**Affiliations:** 1 Department of Biology, Science and Research Branch, Islamic Azad University, Tehran, Iran; 2 Farhangian University, Shahid Mofatteh Teacher Education Paradise, Tehran, Iran; 3 Department of Biology, Faculty of Science, Yazd University, Yazd, Iran; 4 Department of Molecular Medicine, School of Advanced Technologies in Medicine, Tehran University of Medical Sciences, Tehran, Iran; 5 Department of Medical Biotechnology, School of Medicine, Zanjan University of Medical Sciences, Zanjan, Iran; 6 Medical Genetic Center, Endocrinology and Metabolism Research Institute (EMRI), Tehran University of Medical Sciences (TUMS), Tehran, Iran

**Keywords:** Pro12Ala polymorphism, PPAR­γ2 gene, NAFLD, Type 2 diabetes mellitus

## Abstract

**Background:**

The peroxisome proliferator-activated receptors (PPARs) are ligand-activated transcription factors that belong to the nuclear hormone receptor superfamily. Several studies have demonstrated a significant association between Pro12Ala polymorphism of the PPAR­γ2 gene and metabolic disorders. Therefore, this study aimed to evaluate the association of Pro12Ala polymorphism with increased risk of NAFLD in Iranian patients with type 2 diabetes mellitus.

**Methods:**

This cross-sectional study was performed on 145 healthy control subjects and 145 NAFLD patients with a history of type 2 diabetes. Pro12Ala polymorphism genotyping was performed using PCR–restriction fragment length polymorphism (RFLP) technique with the *Bs1I* restriction enzyme.

**Results:**

Our results demonstrated that CC and GG genotypes of Pro12Ala were found in the participants, but there was no statistically significant difference between NAFLD patients and healthy controls (*P* = 0.64 and χ^2^ = 0.21).

**Conclusion:**

This study suggests that Pro12Ala polymorphism of the PPAR­γ2 gene cannot be considered as a risk factor for NAFLD in the Iranian population.

## Introduction

Nonalcoholic fatty liver disease (NAFLD) is one of the most prevalent forms of progressive liver disease, which is characterized by obesity, dyslipidemia, type 2 diabetes mellitus (T2DM), hypercholesterolemia, hypertension, insulin resistance, cirrhosis, liver failure and hepatocellular carcinoma [1–3]. NAFLD is highly prevalent in T2DM and the prevalence of NAFLD in obese adults with T2DM has been estimated to be greater than 70%. The most important treatment method of NAFLD is to increase insulin sensitivity by lifestyle changes [2–10]. Epidemiological, familial, and twin studies have suggested that genetic background and environmental factors play important roles in the pathogenesis of NAFLD as multifactorial diseases [8–16].

The peroxisome proliferator-activated receptors (PPARs) are ligand-activated transcription factors which belong to the nuclear hormone receptor superfamily and consist of three subtypes, each of them encoded by different genes: PPARα (NR1C1), PPARγ (NR1C3) and PPARδ (NP1C2) [3, 16–18]. The PPARγ receptor plays a key role in the regulation of adipocyte-specific genes, lipid metabolism, glucose homeostasis, insulin sensitivity and blood pressure [2, 16, 19, 20]. The human PPARγ gene, located on chromosome 3p25, has nine exons (A1, A2, B, exons 1–6 from 5′ to 3′ direction), and its protein product has three isoforms, PPARγ1, PPAR-γ2 and PPARγ3, which are produced by mRNA alternative splicing of PPARγ [4, 20, 21]. Among them, the PPAR-γ2 isoform is expressed predominantly in the adipose tissue, where it plays an important role in regulating adipogenic differentiation and as a mediator of insulin sensitivity [20, 22–25]. The single nucleotide polymorphisms (SNPs) of the PPAR-γ2 gene have been shown to be associated with susceptibility to several metabolic disorders and many studies have shown a strong relationship between this gene and the occurrence of T2DM in many populations [9, 10, 26, 27]. The polymorphism rs1801282 (c.34C > G) on codon 12 of exon B of the PPAR-γ2 gene, which leads to the substitution of proline with alanine (Pro12Ala), was found to be associated with higher insulin sensitivity, lower body mass index (BMI), decreased risk of T2DM and diabetic nephropathy [28–32]. Several investigations have shown that there is an association between this polymorphism and increased risk of metabolic disorders in various populations. However, published results on the genetic associations of this SNP with NAFLD are controversial and inconclusive [33–36]. This study aimed to elucidate the association of Pro12Ala with increased risk of NAFLD in Iranian patients with type 2 diabetes mellitus as a common metabolic disorder.

## Material and methods

### Subjects

This case–control study was based on 290 individuals of Iranian ancestry. The patient group included 145 patients with biopsy-proven NAFLD with a history of type 2 diabetes attending the Shahid Rajaei Cardiovascular Medical and Research Center in Tehran, Iran. All patients were recruited by use of liver biopsy. Only patients with a negative history of alcohol consumption and other known causes of chronic liver disease (e.g. autoimmune hepatitis, viral hepatitis, use of hepatotoxic medications such as antibiotics, glucocorticoids, tamoxifen or other anti-neoplastic drugs) were analyzed [5, 37]. Patients with unavailable liver ultrasound examination were excluded from the study. The controls were 145 individuals representing a general population sample from the same geographical region who had normal abdominal ultrasonography. Participants were matched for sex and age and numbers of men and women were equal in both groups. All participants gave written informed consent, and this study conforms to the principles of the Declaration of Helsinki. Both patient and control subjects’ characteristics including gender and age are shown in Table 1.

**Table 1:** Characteristics of study population

Characteristics	Patients	Controls	*P* value
Total	145	145	–
Men	73	73	1.0
Women	72	72	
Mean age	53.9±9	51.3±10	0.98

Clinical and laboratory findings were collected for each NAFLD patient from the clinical charts, including fasting blood sugar (FBS), BMI, creatine (Cr), total serum cholesterol (TC), triglyceride (TG), low-density lipoproteins (LDL) cholesterol, high-density lipoproteins (HDL) cholesterol, hemoglobin A1c (HbA1c), and microalbumin.

### DNA extraction and molecular genotyping

Genomic DNA was isolated from peripheral blood leukocytes using Bioneer’s DNA extraction kit (Bioneer, Daejeon, Korea). Pro12Ala (CCG-GCG) alleles were determined by PCR–restriction fragment length polymorphism (RFLP) analysis using *Bs1I* restriction enzyme.

Forward and reverse primer sequences were 5′- TGTCTTGACTCATGGGTGTATTC-3′ and 5′- ATCAGTGAAGGAACCGCTTT-3′, respectively. Each PCR was performed in a final volume of 25 μl, including 100 ng of genomic DNA, 0.5 μl of each primer (10 pmol), 0.5 μl of dNTP, 0.8 μl of MgCl_2_, 2.5 μl of 10x PCR buffer and 2 units of *Taq* polymerase. The PCR conditions were as follows: initial denaturation at 95 °C for 5 min, then 35 cycles of 95 °C for 30 s, annealing at 57 °C for 30 s, extension at 72 °C for 30 s. This was followed by final extension at 72 °C for 5 min. The 185 bp amplified fragment was digested by restriction endonuclease *Bs1I* (Fermentas). The restriction fragments were separated by electrophoresis on a 2% agarose gel. Gel was stained with ethidium bromide and photographed by UV photography.

### Statistical analysis

The data were analyzed using the SPSS 12.0 statistical software (SPSS, Chicago, IL, USA). Using the χ^2^ test, genotype and allele frequencies in the NAFLD and control subjects were compared. Student’s t test was applied to detect differences between groups with various genotypes regarding other variables such as age, FBS TG, TC, LDL, HDL and HbA1c. Odds ratios (OR) and their 95% confidence intervals (CI) were calculated. The associations between Pro12Ala polymorphism in the PPAR­γ2 gene with NAFLD and clinical and biochemical variables were evaluated. Data are presented as mean ± standard deviation (SD) and *P* values of less than 0.05 were considered significant for all tests.

## Results

**Fig. 1:**
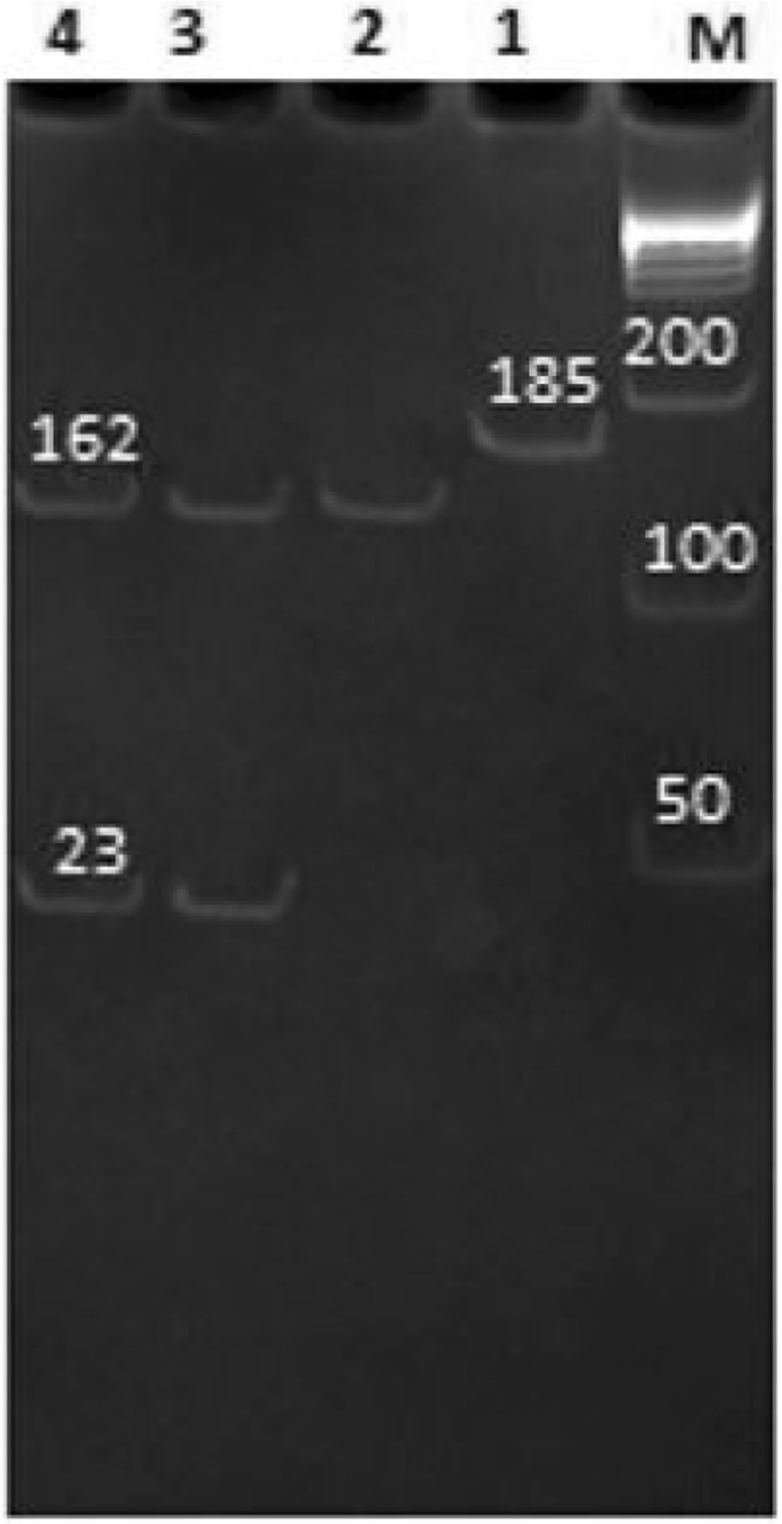
RFLP detection of Pro12Ala polymorphism of PPAR­γ2 gene using gel electrophoresis. M: Ladder 50 bp, lane 1: GG genotype (Pro12Ala) (185 bp), lanes 2, 3, and 4: CC genotype (Pro12Pro) (162, 23 bp)

Digestion of PCR amplifications of the PPAR­γ2 gene with *Bs1I* gave different patterns, as shown in Fig. 1.

The allelic and genotypic frequencies of the Pro12Ala PPAR­γ2 gene SNPs in patient and control subjects are shown in Table 2. As presented, there is no statistically significant difference regarding the allelic and genotypic frequencies of Pro12Ala PPAR­γ2 gene SNPs between the NAFLD patients and healthy control subjects (*P* = 0.64 and χ^2^ = 0.21).

**Table 2:** Genotypic association of the Pro12Ala polymorphism with NAFLD

Variable	Genotype		OR (95% CI)	*P*-value
	CC N (%)	GG (%)		
NAFLD patients	87 (60%)	58 (40%)	1.92 (0.742–1.917)	0.468
Control subjects	93 (64.1%)	52 (35.9%)		
	Alleles		OR (95% CI)	*P*-value
	C N (%)	G N (%)		
NAFLD patients	174 (80%)	116 (20%)	1.192 (0.852–1.668)	0.304
Control subjects	186 (82%)	104 (18%)		

The results showed no significant difference between the Pro12Ala carriers and wild-type individuals in the biochemical variables including BMI, FBS, Cr, lipid profile (TG, TC, HDL-C, and LDL-C), HbA1c, and micro albumin levels (data not shown).

## Discussion

In this study, the association of PPAR­γ2 gene SNP Pro12Ala and increased risk of NAFLD was evaluated in patients with type 2 diabetes mellitus. Our results demonstrate that the polymorphism rs1801282 (c.34C > G, Pro12Ala) in the PPAR­γ2 gene is not associated with increased susceptibility to NAFLD in the Iranian population. Therefore, SNP Pro12Ala cannot be a genetic marker of NAFLD in this population.

NAFLD is independently associated with T2DM, obesity and metabolic syndrome, and the accumulation of fatty acid in the liver impairs insulin sensitivity. It has been hypothesized that the genetic variations (SNPs) found in metabolic syndrome patients may be related to NAFLD [38–41]. PPARγ is involved in regulation of adipogenic differentiation, lipid metabolism, insulin sensitivity and hepatic steatosis [42]. Thus, PPARγ is a promising candidate gene for several metabolic syndromes including T2DM, obesity, and NAFLD. However, the effect of PPARγ gene SNPs on the pathogenesis of NAFLD has not been clearly documented. Therefore, investigating the relationship between Pro12Ala PPAR­γ2 gene SNPs and increased susceptibility to NAFLD is important.

According to our results, the Pro12Ala SNP does not induce NAFLD in the Iranian population. Since the PPAR­γ gene contains several polymorphisms, other genetic variations may affect expression of the related molecule and be associated with NAFLD, which needs to be evaluated by further investigations.

However, the associations of Pro12Ala polymorphism with NAFLD have been reported to be varied in different populations and ethnic groups. For example, Gupta et al. investigated the Pro12Ala variant in 98 NAFLD patients and 280 matched controls and found that the Pro12Ala polymorphism was significantly associated with NAFLD in an Indian population [16]. Gawrieh et al., by evaluation of an American population, demonstrated that the SNP was associated with histologically advanced NAFLD [43]. Yang et al. revealed that Pro/Pro genotype of Pro12Ala was an increased risk factor for the development of NAFLD in Chinese patients [44]. A meta-analysis also demonstrated that the Pro12Ala variation of PPAR­γ2 was associated with susceptibility to NAFLD in East Asians, but not in European populations [13]. Bhatt et al. in a study with 162 Asian NAFLD patients and 173 controls observed that Pro12Ala SNP not only was associated with NAFLD but also was associated with alkaline phosphatase, higher serum TG, and waist-hip ratio [45].

Although the mentioned investigations revealed that PPARγ gene polymorphism was significantly associated with NAFLD in some population, Dongiovanni et al. observed that the PPAR-γ2 Pro12Ala frequency was not significantly different between NAFLD patients and healthy controls, in a study enrolling 202 Italian patients and 346 matched controls [12]. Rey et al. also reported that the incidence of the Ala12 mutant in German patients with NAFLD was not significantly different from healthy controls, and no significant difference was observed in histological inflammation in NAFLD [46]. Cao et al. studied the effect of Pro12Ala PPAR­γ2 gene SNPs in Chinese patients with NAFLD, including 169 NAFLD patients and 699 healthy subjects. Their analysis showed that the allele frequency was not significantly different between the two groups (*p* > 0.05) [4]. Domenici et al. found that Pro12Ala polymorphism was not associated with clinical, laboratory and histological parameters in NAFLD patients [8]. In the study by Chen et al., no association was found between PPAR-γ2 Pro12Ala substitution and NAFLD, and no significant difference was observed between this polymorphism and physiological variables [2]. Results from the meta-analysis of Sahebkar et al. indicated that Pro12Ala variation of PPAR­γ2 was not associated with NAFLD risk [47].

Generally, it seems that there is a significant controversy regarding the roles of PPARγ gene polymorphism in NAFLD pathogenesis. These consistencies could be the result of the different ethnic structure, referral and ascertainment biases, inadequate sample size, lack of mediators’ adjustment, or publication bias.

A possible limitation of our study was the small sample size, so further larger studies are required for confirmation.

In conclusion, the results of this study showed that Pro12Ala polymorphism of the PPAR­γ2 gene is not associated with increased susceptibility to NAFLD in the Iranian population. However, more studies with a larger sample size are needed to confirm this.

## Abbreviations

NAFLD: Non-alcoholic fatty liver disease
PPARs: Peroxisome proliferator-activated receptors
RFLP: Restriction fragment length polymorphism
T2DM: Type 2 diabetes mellitus
